# Chemical Alternatives Assessment: Progress and Perspectives in Hazard Characterization, Exposure Assessment, and Life Cycle Assessment

**DOI:** 10.3390/jox16060195

**Published:** 2026-10-13

**Authors:** Yipeng Lu, Xiaoqian Chen, Pin Gao

**Affiliations:** 1 College of Environmental Science and Engineering, Donghua University, Shanghai 201620, China; 2 Shanghai Academy of Public Measurement Co., Ltd., Shanghai 201203, China

**Keywords:** chemical alternatives, hazard characterization, exposure assessment, life cycle assessment

## Abstract

With rapid technological advancement and accelerating industrialization, legacy chemicals and emerging contaminants have caused increasingly severe adverse impacts on ecological environments and human health due to their persistence, bioaccumulation, and toxicity. Developing and scaling up green chemical alternatives is a critical pathway to mitigate exposure risks from conventional hazardous substances and achieve sustainable industrial development. However, to prevent a recurring “pollution-substitution-repollution” cycle, often termed “regrettable substitution”, there is an urgent need to establish a scientifically sound and forward-looking chemical alternatives assessment (CAA) framework. This work presents a systematic overview of the evolution of global CAA guidelines and provides an in-depth analysis of recent research advances in assessment methodologies across three core domains, including hazard characterization, exposure assessment, and life cycle assessment (LCA). We highlight that modern CAA is undergoing a profound paradigm shift. Hazard characterization is transitioning from traditional in vivo experiments to predictive toxicology, anchored in new approach methodologies; exposure assessment is evolving from static, conservative assumptions to refined, dynamic coupled multi-media models; and LCA is expanding from end-of-pipe environmental impact auditing to source-level interventions that integrate prospective prediction and green design. By connecting insights across scales, from microscopic molecular mechanisms to macroscopic ecosystem-level outcomes, this study aims to provide relatively comprehensive methodological guidance for chemical alternative assessments. In turn, this work provides both the theoretical foundation for “Safe and Sustainable by Design (SSbD)” chemical development and the practical reference for establishing robust scientific toolkits for prospective chemical classification and regulatory decision-making.

## 1. Introduction

**Figure 1:**
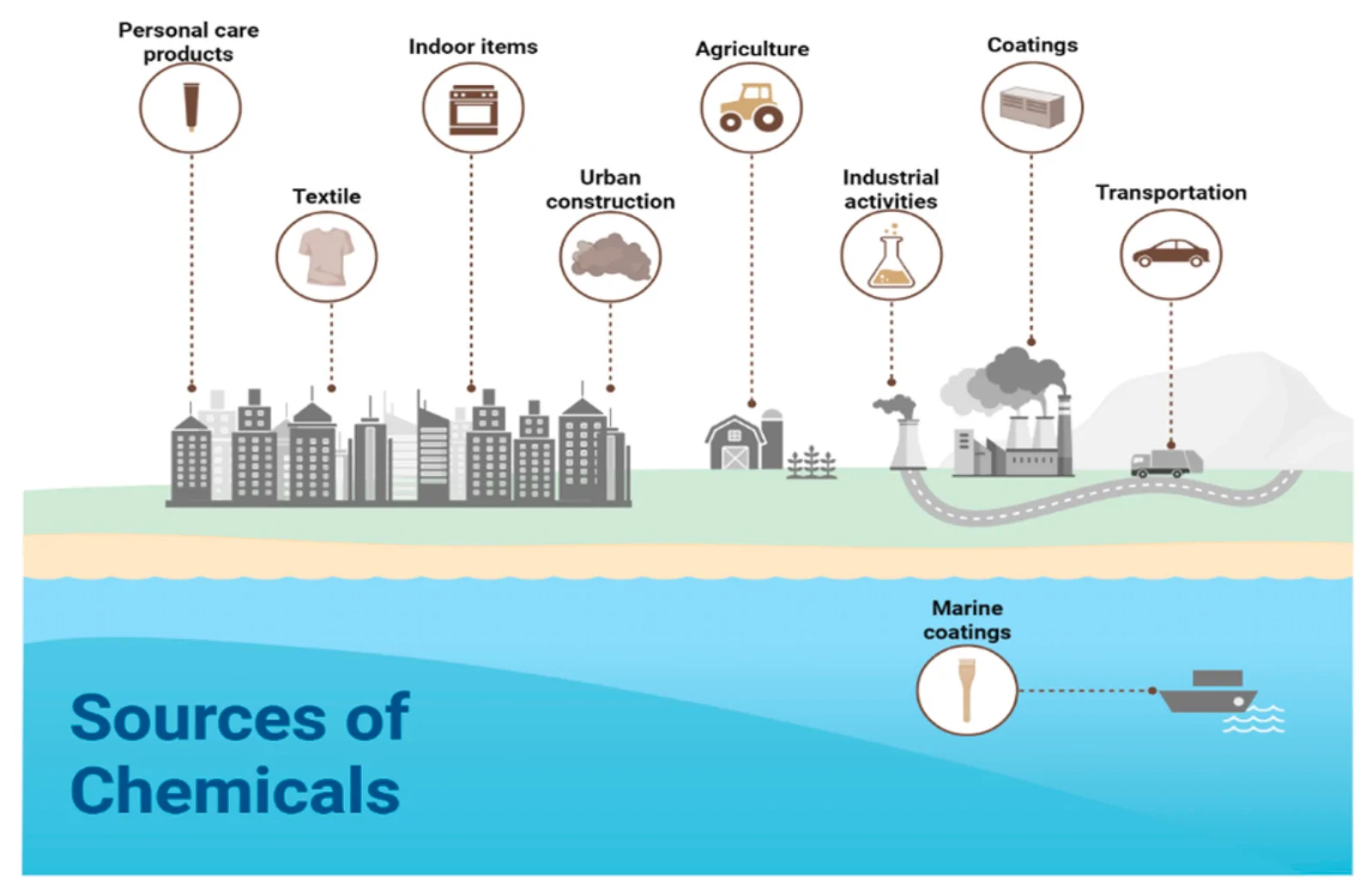
Source of chemicals (created by BioRender).

**Figure 2:**
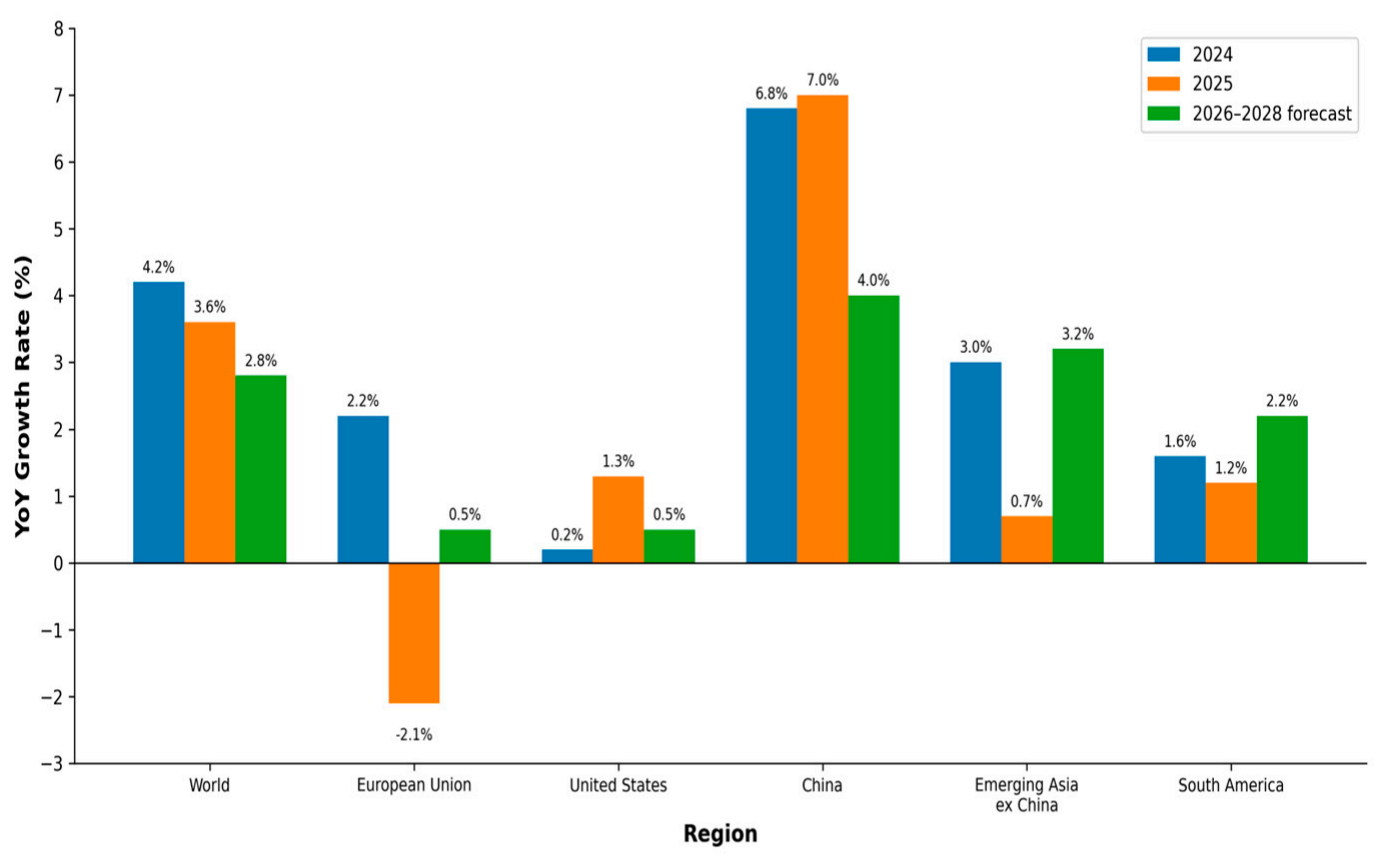
YoY growth rate in global chemical industries (excluding pharmaceuticals). The data are taken from the chemicals industry report in the *2025 BASF Report* [2].

**Figure 3:**
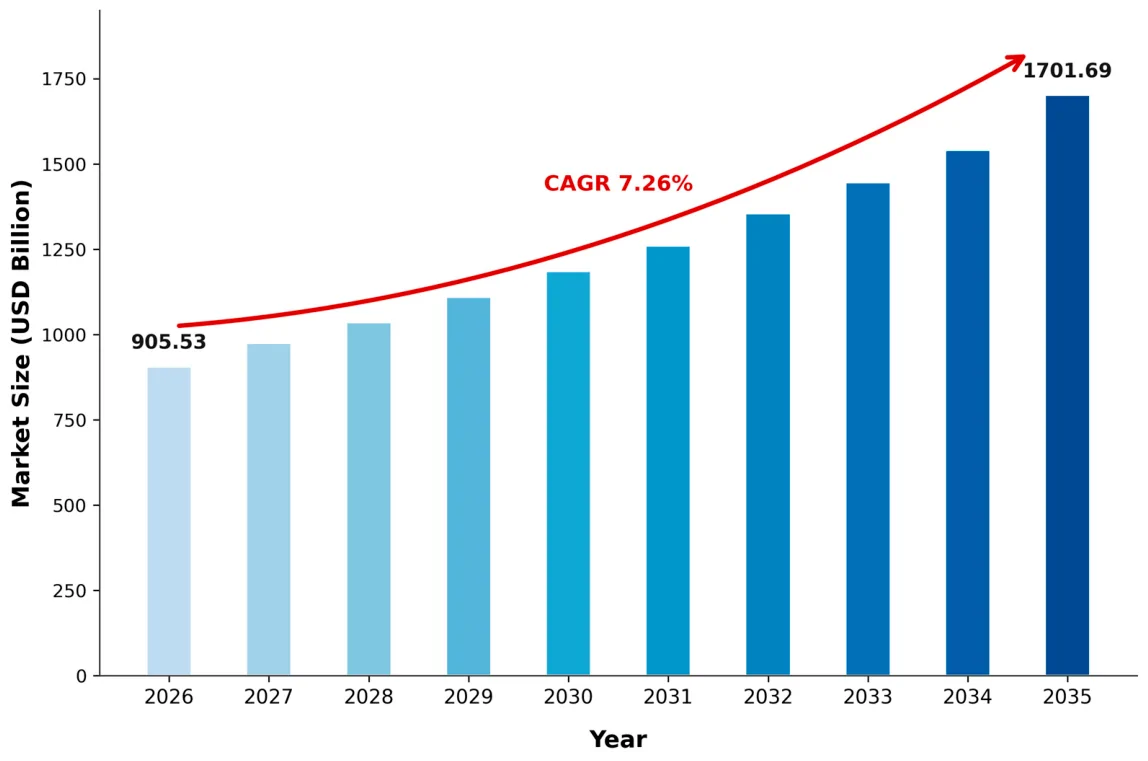
Global commodity chemicals market size, 2026–2035 (USD billion).

Chemicals form the cornerstone of modern material civilization and play an irreplaceable role in virtually every aspect of daily life, from supporting basic human survival to improving quality of life and from safeguarding public health to advancing cutting-edge technology [1]. Concurrently, driven by the diverse demands of modern society (Figure 1), the variety, application scope, and production volume of chemicals have all expanded exponentially (Figure 2) [2]. According to the *Deloitte/Federal Reserve Report*, global chemical production will increase by 3.5% in 2025. Among them, semiconductor, plastic, and rubber are the fastest-growing end-use industries. The global chemical industry achieved a total output value of approximately 5.8 trillion US dollars in 2025, with a year-on-year growth of 3.2%. Research on the global chemical market indicates that by 2026, the global market for bulk chemicals will reach 905.53 billion US dollars, with significant growth expected. By 2035, it is projected to reach 1701.69 billion US dollars. During the forecast period from 2026 to 2035, the market is expected to have a compound annual growth rate of 7.26% (Figure 3). During this period of rapid growth, historical mismanagement and improper disposal practices have led to the release of large quantities of hazardous industrial chemicals into ecosystems via environmental media, including air, water, and soil. Once introduced into the environment, these pollutants cycle through all trophic levels of ecosystems, undergo biomagnification along the food chain, and ultimately enter the human body as well as the bodies of plants and animals through multiple exposure routes, posing severe threats to both public health and ecological security [3].

In view of these challenges, enterprises and research institutions are actively exploring and developing less hazardous chemicals as alternatives to conventional products to address environmental and human health risks and meet the demands of industrial and societal development. Against this backdrop, the concept of “substitution” has been progressively integrated into chemical management frameworks worldwide [4]. This paradigm shift has driven the promulgation of diverse policies, stringent regulatory requirements, restrictive source-control measures, and industry-led sustainable chemistry initiatives, all aligned toward restricting or phasing out the production and use of specific hazardous chemicals. However, owing to the early lack of comprehensive and scientifically rigorous evaluation mechanisms for chemical alternatives, many so-called “environmentally friendly” alternatives have inadvertently locked chemical management into the vicious cycle of “regrettable substitution”. A classic illustration of this dilemma is the 1987 Montreal Protocol: while the agreement successfully restricted the use of chlorofluorocarbons and effectively halted ozone layer depletion, their primary replacement, hydrofluorocarbons, has emerged as a potent greenhouse gas that substantially exacerbates global warming [5,6]. When alternatives are not truly “environmentally friendly”, they not only spark public concern but also erode societal trust in green innovation, leading to widespread skepticism toward alternatives marketed as “green”. To prevent similar cases of “regrettable substitution” from recurring [7,8], it is necessary to establish a mature and comprehensive alternative evaluation mechanism to ensure that alternatives are safer without compromising product performance, thereby protecting human health and ecological security. To date, Western developed countries, particularly the United States and the European region, along with their governmental agencies and non-governmental organizations, have developed technical guidelines for alternative assessment, with clear specifications for relevant technical methods and requirements.

Against this background, this review systematically analyzes the literature published over the past decade by combining core keywords, including “chemical alternatives”, “human health”, “environmental hazards”, “life cycle assessment”, and “exposure assessment”, and comprehensively synthesizes current methodologies and mainstream assessment frameworks for chemical alternatives. Unlike studies that focus on a single substance or a single methodological module, this paper begins with the evolution of the CAA framework, systematically reviews the main assessment methods involved in the framework, and attempts to integrate the three dimensions of “hazard characterization—exposure assessment—LCA” from a systems perspective. The analysis progresses from micro-level toxicological mechanisms to meso-level exposure models and macro-level life cycle boundaries, whilst exploring potential links with strategic directions such as SSbD and planetary boundaries. The contribution of this paper lies not in proposing new technical methods, but in providing a relatively comprehensive, integrated perspective for the induction and analysis of CAA methodology. This review provides a theoretical foundation and robust technical references, with the overarching goal of strengthening alternatives assessment protocols for hazardous substances, improving ecological risk management practices, and accelerating the proactive research and industrial application of green alternatives.

## 2. Chemical Alternative Assessment Frameworks: Global Evolution and Development

**Figure 4:**
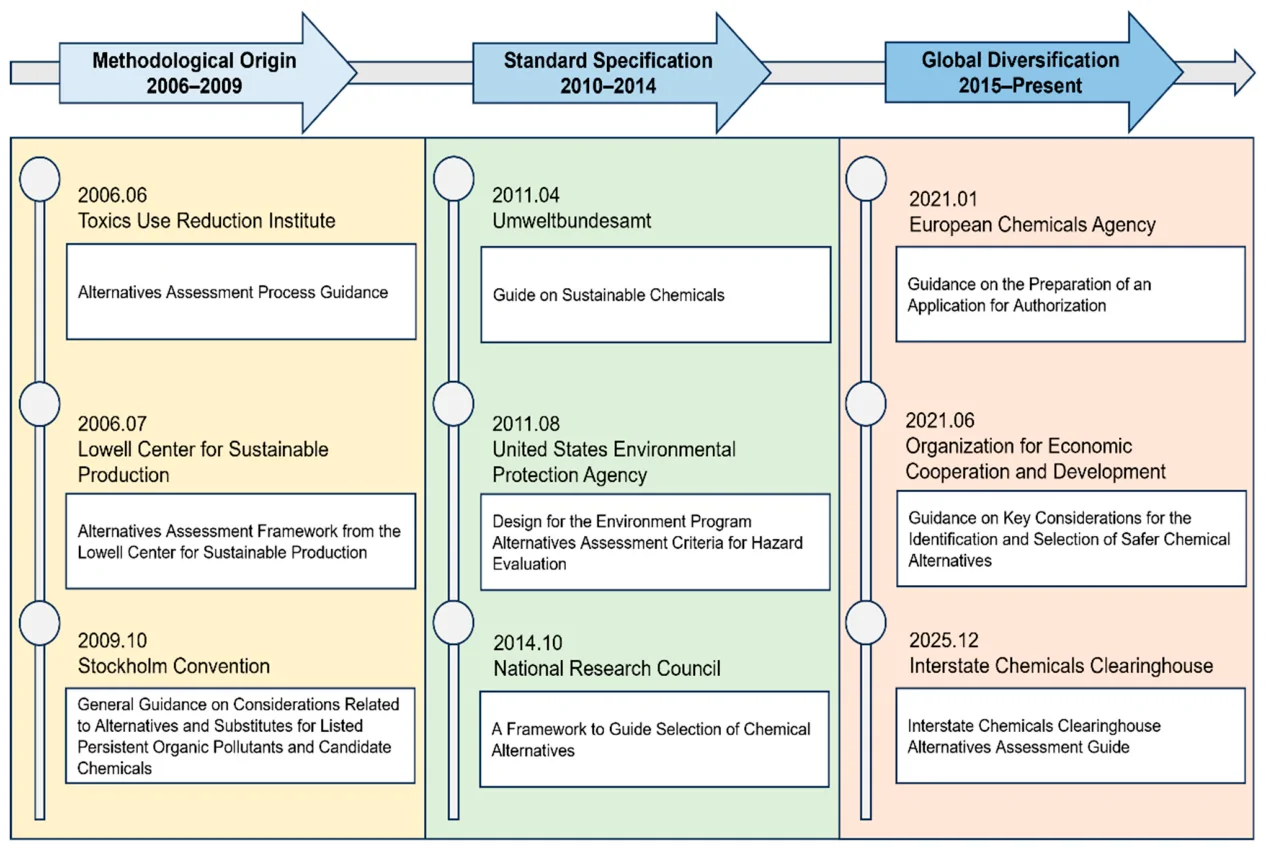
Evolution of chemical alternatives assessment frameworks.

Tracing its origins, the U.S. Environmental Protection Agency (EPA) officially launched the “Significant New Alternatives Policy” program in 1994 under the authority of the Clean Air Act, marking the first establishment of chemical alternatives assessment (CAA) as a systematic national-level framework [9]. The core objective of this program is to identify substances that present lower risks to human health and the environment via scientific assessment, thereby regulating the phase-out and replacement process of ozone-depleting substances. Notably, the program formalized the “acceptable risk” principle, which recognizes that alternative substances are not required to be completely risk-free. Although this pragmatic approach laid the conceptual foundation for subsequent “suboptimal alternative” frameworks to some degree, its core historical significance lies in shifting alternative assessments from a voluntary corporate initiative to a binding national regulatory requirement, thus establishing an early foundational model for the development of the global CAA system. Since this milestone, alternative assessment frameworks developed by the international community have become increasingly comprehensive and refined; the overall development trajectory and key mainstream guidelines are summarized in Figure 4 and Table 1.

During the early exploration and method development phase, the Toxics Use Reduction Institute (TURI) at the University of Massachusetts Lowell published the *Alternatives Assessment Process Guidance* in June 2006. This pioneering guidance first incorporated multi-indicator matrices and safety-tier determination into the CAA framework, and evaluated five chemicals of great concern, such as lead, formaldehyde, perchloroethylene, hexavalent chromium, and di(2-ethylhexyl) phthalate (DEHP) [10]. Subsequently, the Lowell Center for Sustainable Production proposed its own Alternatives Assessment Framework, which further expanded the scope of evaluation by emphasizing the paradigms of closed-loop recycling and material circularity [11]. In October 2009, the Stockholm Convention formally incorporated alternatives analysis into international legally binding treaty text for the first time, granting it international legal force. Following this milestone, the United Nations Environment Programme (UNEP) released corresponding guiding principles and established standardized screening criteria for the replacement of persistent organic pollutants [12]. A core academic consensus that emerged during this period was that alternatives analysis delivers greater precautionary value than standalone risk assessment. This consensus drove a fundamental paradigm shift in chemical governance, shifting from “end-of-pipe” emission control to source prevention. It thereby overcame the limitations of traditional risk management, which focused heavily on “how to safely manage toxic exposure”, and oriented the field toward the pursuit of “inherent safety” that can be the complete elimination of intrinsic chemical hazards at the source. Since 2010, driven by regulatory attention and policy intervention, CAA has entered a stage of rapid development and standard refinement. The United Nations, along with European and North American countries, began collaborative research and released relatively comprehensive assessment frameworks, methods, and standardized systems. In 2011, the Umweltbundesamt (UBA) published the *Guide on Sustainable Chemicals*, which expanded the assessment scope to incorporate sustainability dimensions, including climate impact and resource efficiency [13]. In the same year, the U.S. EPA established specific classification criteria for chemical hazards based on toxicological data in its “Design for the Environment Program Alternatives Assessment Criteria for Hazard Evaluation” [14].

In response to the growing complexity of chemical applications, the international community has increasingly called for assessment methodologies that are “consistent”, “scientifically rigorous”, and “globally applicable”. Accordingly, in October 2014, the U.S. National Research Council released “A Framework to Guide Selection of Chemical Alternatives”. This framework established a highly systematic 13-step assessment model and has since been widely recognized as the “gold standard” in the CAA field [15,20]. Most importantly, it was the first work to systematically propose a methodological logic for addressing data gaps and supporting multi-criteria decision-making, with a specific focus on resolving the complex trade-offs between environmental benefits and public health risks. In recent years, the CAA framework has evolved in depth to meet the requirements of differentiated regulation and diversified application scenarios. In 2021, the European Chemicals Agency (ECHA) updated its “Guidance on the Preparation of Applications for Authorization”. The regulation explicitly requires that for substances of very high concern approaching their “sunset dates”, enterprises applying for derogation or exemptions for continued production and use must submit a sound and comprehensive analysis of alternatives [16]. Concurrently, the Organization for Economic Co-operation and Development (OECD) released “Guidance on Key Considerations for the Identification and Selection of Safer Chemical Alternatives”. The core goal of this guidance is to resolve long-standing ambiguity surrounding the definition of “safety” and to establish minimum benchmark requirements for identifying safer chemical alternatives, applicable regardless of the assessing entity or specific assessment framework adopted [17]. In China, to synergistically advance “emerging pollutant control” and “collaborative pollution reduction and carbon mitigation, the Chinese Society for Environmental Sciences (CSES) released the “Technical Guidelines for the Assessment of Green Alternatives” in 2024. Drawing on international best practices, this document establishes a green hierarchical assessment method based on the inherent hazards of chemical substances, providing a standardized basis for screening safer alternatives in China [18]. Additionally, the U.S. Inter-State Chemical Clearance Center (IC2) released Version 1.2 of its “Guidelines for Substitute Assessment” [19]. Its “modular” assessment framework offers outstanding flexibility and operational practicability, which has had a far-reaching impact on the application of CAA by enterprises across different industrial scales.

## 3. Hazard Assessment: Perspectives and Research Trends

### 3.1. Screening of Key Assessment Perspectives

A review of the major CAA frameworks outlined above (Table 2) demonstrates that the adoption frequency and weighting of distinct assessment perspectives follow a clear hierarchical progression. “Human health hazards” and “environmental hazards” are required universal metrics across all evaluated frameworks, reflecting the core, gatekeeping role of hazard characterization in the initial screening of chemical alternatives. Secondary to hazard assessment, “exposure assessment” has been integrated into the vast majority of existing frameworks, indicating a fundamental paradigm shift in modern evaluation philosophy from standalone intrinsic hazard identification to comprehensive risk quantification that accounts for realistic exposure scenarios. In contrast, physicochemical properties and LCA are currently required as mandatory endpoints in only approximately half of the high-quality frameworks (e.g., ECHA and IC2). These two dimensions address distinct assessment needs: the physicochemical properties primarily serve to preempt physical hazards during chemical production, while LCA tracks the full holistic environmental footprint from a cradle-to-grave perspective. In many cases, physicochemical properties only function as a supplementary technical input for other assessment modules.

The horizontal comparison unmasks a fundamental paradigm cleavage: while frameworks like EPA’s DfE operate as strictly hazard-centric filtering tools completely decoupled from exposure tracking, global treaty-level guidelines (e.g., UNEP and ECHA) mandate varying degrees of life cycle thinking and conditional exposure mapping to avoid cross-media pollution shifting. This discrepancy not only reflects differences in stringency and scope across different guidelines, but also highlights the irreversible trajectory of chemical alternatives assessment toward life-cycle-based, multi-dimensional, and integrated evaluation.

### 3.2. Research Methods and Bibliometric Analyses

**Figure 5:**
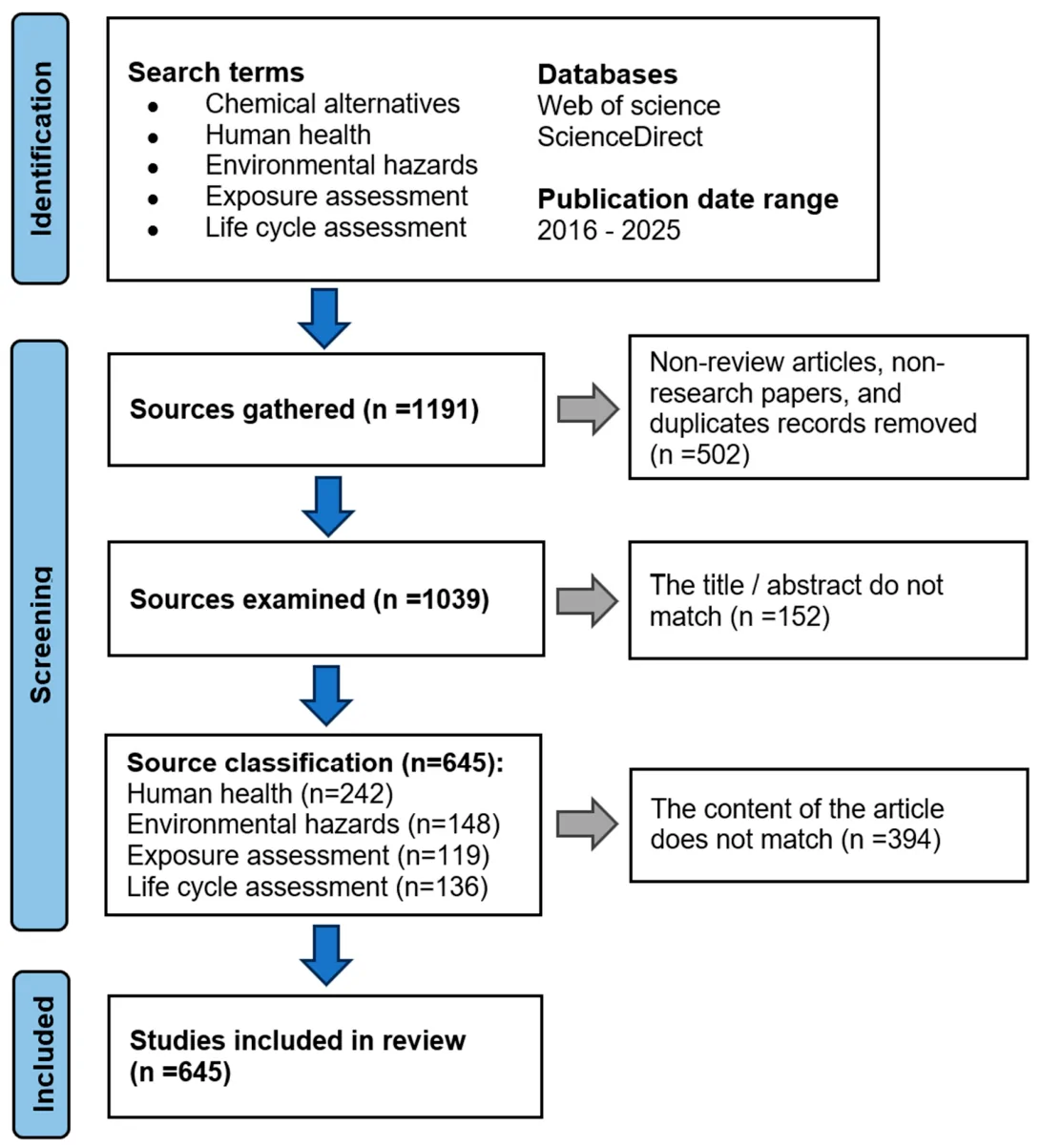
Flowchart for literature screening based on PRISMA.

**Figure 6:**
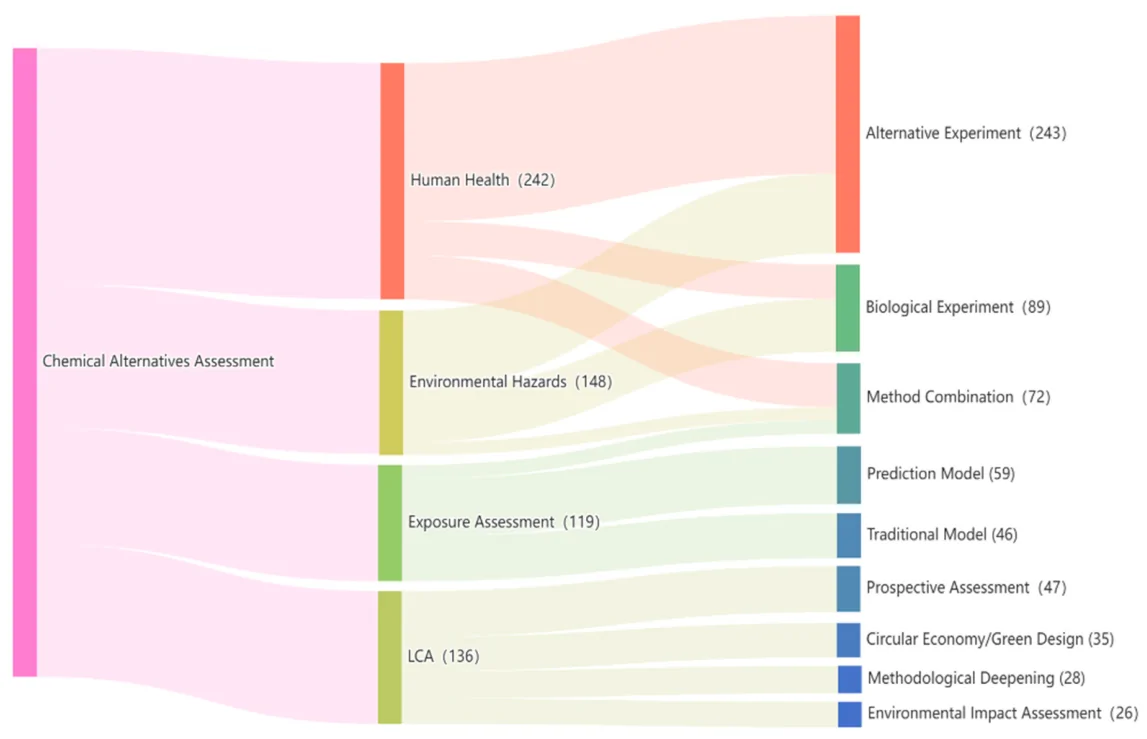
Literature distribution for chemical alternatives assessment (recent decade).

**Figure 7:**
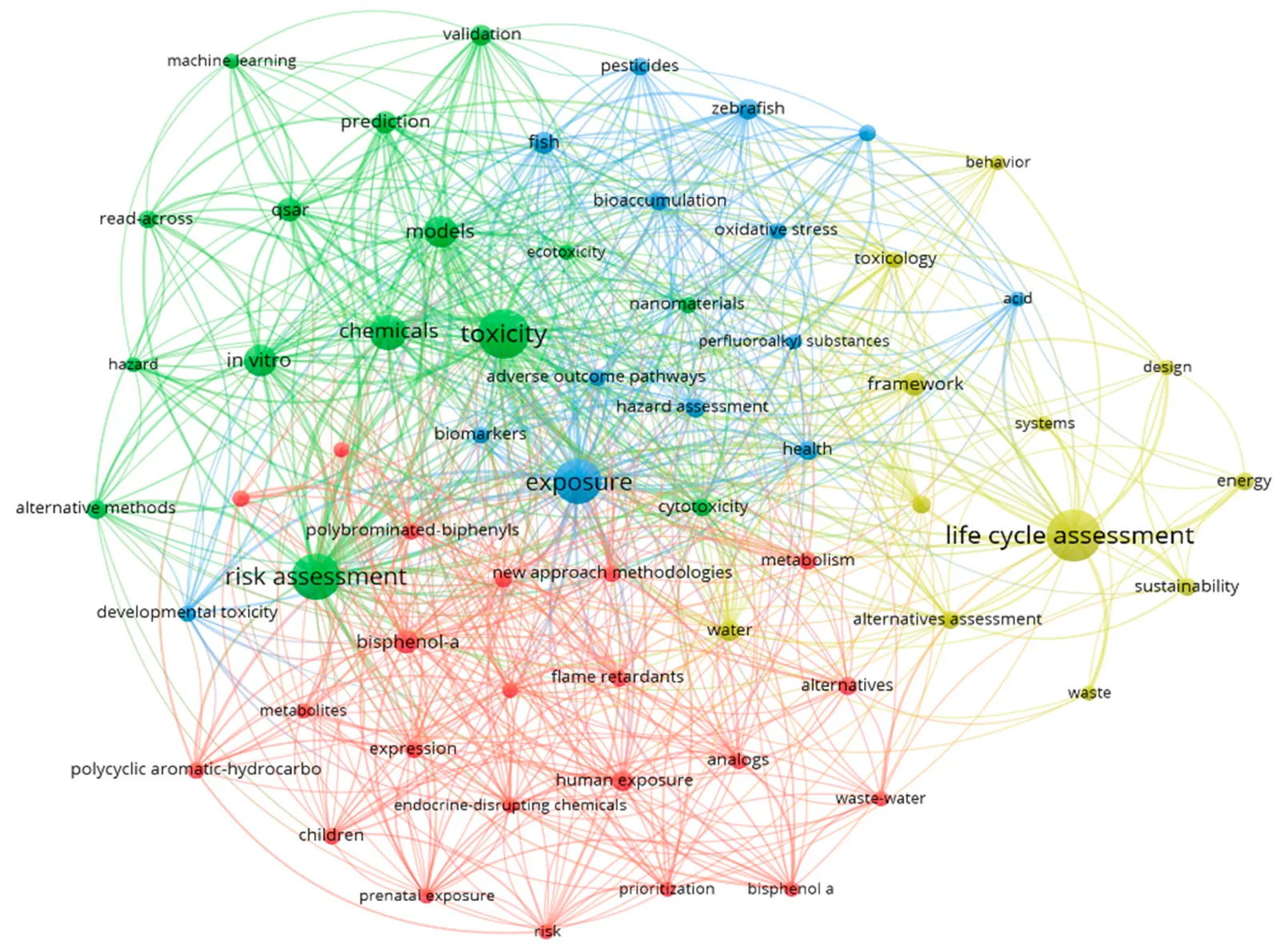
Keyword analysis mapping of included studies.

To further clarify research hotspots and evolutionary trends in the CAA field over recent years, this study retrieved relevant literature published over the past decade from the ScienceDirect and Web of Science databases (Figure 5). Following manual review, screening, and curation of the retrieved research records, bibliometric analysis was conducted, and the results are presented in Figure 6 (data derived from categorized statistics in the literature) and Figure 7 (generated using VOSviewer 1.6.20, where “Co-occurrence” was selected for “Type of analysis”, “All keywords” for “Unit of analysis”, the “Minimum number of occurrences of a keyword” was set to 10, “Association strength” was selected for “Normalization Method”, and keywords of the same type were merged).

As intuitively demonstrated by the Sankey diagram (Figure 6), “human health” remains the primary research priority in this field, which has directly driven extensive research and development of safer chemical alternatives. Concurrently, the share of research focused on environmental hazards, LCA, and exposure assessment is highly comparable across these themes, indicating that the field’s evaluation paradigm has shifted from fragmented reliance on isolated toxicity metrics to holistic scrutiny across the entire chemical life cycle. Additionally, the number of publications focused on in vitro and in silico alternative methods is substantially higher than the number focused on traditional in vivo biological assays.

Furthermore, keyword co-occurrence network (Figure 7) analysis identifies four synergistic knowledge clusters: (1) a predictive toxicology framework underpinned by quantitative structure–activity relationships (QSAR), machine learning, and in vitro assays; (2) environmental behavior and ecotoxicity characterization of emerging contaminants; (3) health risk mitigation for sensitive populations (e.g., prenatal and childhood exposure) targeting high-risk endocrine-disrupting chemicals; and (4) LCA-driven green design and circular economy strategies. Overall, the CAA field undergoes a critical paradigm shift. It transits from binary toxicity screening toward multi-dimensional integration of predictive toxicology, source mitigation, and life cycle sustainability optimization, and ultimately supports macro-level integration of human exposure risk assessment and life cycle sustainability assessment. Bridging new approach methodologies (NAMs) with multi-criteria decision-making to balance public health protection and industrial eco-efficiency represents the most critical frontier for future research in this field.

This evolutionary trajectory reflects the continuous deepening of research into microscopic molecular mechanisms, alongside the systematic expansion of evaluation perspectives toward macroscopic green design and systematic management. Within the modern CAA framework, the core objective is to accurately identify and quantify the potential hazards of alternative chemicals to human health and ecological systems so as to completely avoid the occurrence of “regrettable substitution”. Driven by increasingly stringent regulations on laboratory animal welfare and the rapidly growing demand for high-throughput screening, the shift in assessment methodologies from traditional in vivo bioassays to NAMs has become an irreversible development trend. Meanwhile, the requirement for accurate quantification of chemical exposure risks throughout the entire life cycle, coupled with the increasing demand for transparency of models and algorithms [21], is jointly promoting the formation of a green chemistry assessment loop that covers the whole process from conceptual molecular design to end-of-life disposal of chemicals.

## 4. Human Health and Environmental Hazard Characterization

**Figure 8:**
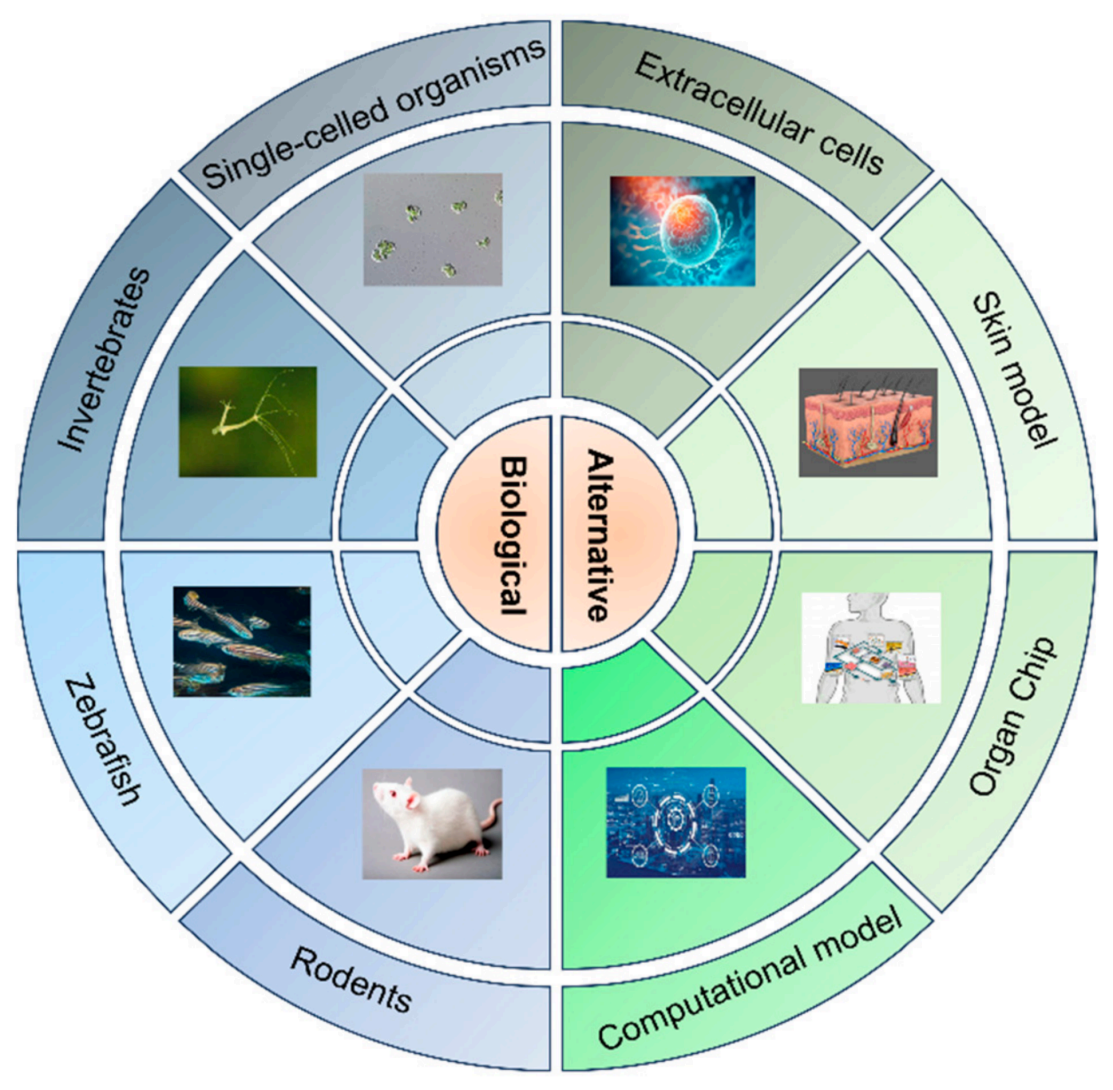
Classification of biological and alternative experiments.

Within the CAA framework, the accuracy of hazard characterization fundamentally determines the reliability of baseline risk assessment. Figure 8 intuitively illustrates the classification nomenclature and evolutionary trajectories of traditional biological approaches versus emerging alternative methodologies for chemical hazard characterization. Critically, the left and right axes of this diagram are not mutually exclusive or diametrically opposed. Instead, they collectively form a complementary continuum shaped by mechanistic mapping, data integration, and ethical advancement. The biological experimental approaches on the left deconstruct toxicity from macroscopic apical endpoints down to microscopic cellular mechanisms. The high-quality phenotypic and mechanistic data generated by these approaches serve as the foundation for training, calibrating, and validating the alternative models positioned on the right. Conversely, the alternative methodologies on the right synergistically integrate in vitro data (obtained from cellular and organotypic models) with in silico simulations (e.g., physiologically based toxicokinetic modeling and advanced machine learning/statistical algorithms), and have successfully addressed longstanding bottlenecks of low throughput and high uncertainty in cross-species extrapolation that are inherent to traditional animal experiments.

Growing societal concern over laboratory animal welfare has also contributed to increasing resistance to direct in vivo animal experimentation [22]. Driven by high-profile public movements such as the European Citizens’ Initiative calling for an end to animal testing, regulatory bodies, including the European Commission, have strategically developed policy roadmaps targeting the full and ultimate replacement of animal-based testing [23]. Concurrently, the U.S. Food and Drug Administration enacted the Modernization Act 2.0, which issued a clear legislative mandate actively supporting the adoption of non-animal alternative methodologies for chemical safety assessment [24,25].

This paradigm shift in chemical safety assessment is driven not only by ethical imperatives but also by fundamental practical needs. Amid rapid expansion of the global chemical industry, traditional animal testing, which is characterized by low-throughput, long experimental cycles, and high operational costs, has long been unable to address the challenges posed by large-scale chemical hazard screening. Consequently, research and development priorities have shifted toward efficient, high-throughput computational prediction models, moving away from the time- and resource-intensive brute-force approaches of the past. More importantly, compared with traditional in vivo animal bioassays, in vitro models such as human stem cell-derived organoids and algorithmic models built on human population big data can directly elucidate human-specific toxicological mechanisms, fundamentally eliminating the well-documented uncertainties associated with interspecies extrapolation. It is against this backdrop that next-generation risk assessment (NGRA), an integrated framework that incorporates multiple NAMs, has gradually emerged as a strategic consensus across academia and industry. Ultimately, the high-precision experimental and predictive data generated by NAMs form an indispensable cornerstone for the construction and refinement of the entire NGRA framework.

### 4.1. Optimization of Traditional Bioassays Leveraging Emerging Technologies

While animal models have intact physiological systems that provide the most intuitive holistic insights into complex biological effects such as reproductive development and chronic target-organ toxicity, and have maintained a dominant position in toxicological research over the past decades, traditional animal experimentation currently faces inevitable obsolescence within modern regulatory science under substantial public consensus and ethical pressure, unless it undergoes aggressive evolution toward high-throughput screening, technological refinement, and animal reduction. To address this paradigm crisis, researchers are developing more representative biomimetic models and advancing miniaturization technologies, successfully establishing an efficient, cost-effective, and ethically compliant evaluative methodology.

The modern evaluative paradigm has expanded beyond single mortality metrics for model organism selection and the integration of multidimensional endpoints to encompass multi-tiered ecological assessment frameworks that incorporate reproductive, morphological, and neurobehavioral endpoints. For example, a multi-trophic monitoring matrix utilizing *Eisenia fetida*, *Enchytraeus crypticus*, *Hypoaspis aculeifer*, and *Folsomia candida* successfully reconciles organic waste resource utilization and soil ecological safety assessment [26]. The combination of the diatom index and *Hydra* regeneration assays has also identified potential teratogenic hazards in freshwater ecosystems [27]. Additionally, *Caenorhabditis elegans* and *Drosophila melanogaster*, which share high genetic homology with humans, have been strategically deployed to investigate the nephrotoxicity of nano-pesticides and heavy metals, respectively [28,29]. Notably, because fish embryos and larvae are exempt from most animal welfare regulatory requirements, they have become key surrogate models to replace mammalian experimentation [30]. When incorporated into joint testing schemes paired with automated live-cell imaging systems or *Caenorhabditis elegans* [31,32], these aquatic models significantly improve screening throughput for developmental neurotoxicity. Focusing on the molecular basis of altered gene expression, researchers have leveraged fathead minnow (*Pimephales promelas*) embryo toxicity assays to develop novel frameworks based on the transcriptomic point of departure, enabling high-precision prediction of whole-organism toxicity thresholds [33]. Although these animal alternative models offer clear advantages in terms of ethics, throughput, and cost, they still suffer from issues such as ‘insufficient tissue specificity’ or an ‘inability to fully extrapolate to chronic human pathologies’ when simulating long-term chronic toxicity or complex multi-organ metabolic kinetics. They therefore require hierarchical integration, multi-species/multi-endpoint validation, and the clarification of their protective thresholds.

These micro- and systemic-level innovations have further promoted experimental approaches toward miniaturization and non-destructive testing. For example, the modified Ames test incorporates human cell S9 extract as a novel metabolic activation system to better simulate human-specific metabolic processes [34]. Moreover, the quantitative image-based screening method for the analysis of images of rat ovarian surfaces eliminates labor-intensive tissue sectioning and greatly streamlines the evaluation process [35]. In summary, whether multidimensional topological expansion of model organism systems or miniaturized refinement of experimental techniques, the core of these advances is the high-efficiency accumulation of high-quality toxicological evidence via high-throughput workflows. This technological consolidation is driving a profound paradigm shift in conventional risk assessment, from the traditional “experiment-driven” approach to an increasingly “data-driven” framework. Following this trajectory, researchers have aggregated historical in vivo and in vitro NOAEL/LOAEL datasets for secondary modeling using advanced benchmark dose (BMD) statistical methods (enter animal toxicity data, BMR = 10%, BMRF = 1; the unilateral 95 per cent lower confidence limit of the BMD is taken as the starting dose, which is then converted to a human equivalent dose and corrected for the uncertainty factor), establishing more scientifically rigorous human health risk benchmarks for bisphenol A (BPA) alternatives [36]. BMD modeling is not “assumption-free”; rather, it seeks to minimize subjective choices by standardizing the BMR, adopting a uniform confidence level, and using AIC for model selection. Overall, this approach, which mathematically models biological responses with high accuracy, substantially reduces dependence on live animal models while strictly maintaining the validity of safety assessments. However, the model form itself remains an empirical mathematical function, and there is still considerable uncertainty in extrapolating results from animals to humans.

### 4.2. Multi-Dimensional Predictive Characterization and Early-Warning Risk Based on NAMs

Under the auspices of the U.S. CAA, the “predictive characterization” of hazards to human health and the environment serves as a core strategy to prevent “regrettable substitution”. The challenges faced by CAA when dealing with tens of thousands of new chemicals for which data are scarce are that traditional experiments cannot meet the required timeliness, making it necessary to rely on predictive toxicology (NAMs) to eliminate hazards at the design stage (SSbD), ensuring that alternative chemicals maintain superior safety profiles across their entire life cycle.

Within the domain of in vitro and organoid-based models, human-derived cellular systems consistently demonstrate greater sensitivity and higher human biological relevance compared to conventional animal-based surrogates. For instance, researchers have employed human bronchial epithelial cells (BEAS-2B) to proactively predict early-stage cellular events preceding carcinogenesis induced by tobacco smoke and particulate matter (PM_2.5_) [37,38]. Anchored in human hepatoma cells (HepG2) and human neurosphere models, investigators have also accurately identified latent metabolic and neurotoxic risks associated with common alternative plasticizers, including BPA alternatives and DEHP [39,40]. Additionally, laboratory-cultured human skin sections have been applied to assess the dermal irritation potential of medical devices and to simulate real-world exposure to dyes on thermal paper [41,42]. Furthermore, electrospun nanofiber membranes have been used to engineer co-culture systems that mimic ocular and pulmonary tissue barriers, enabling detailed investigation of particulate matter toxicity in target tissues [43]. Collectively, these advanced applications clearly demonstrate the unique advantages of in vitro test methods for elucidating complex toxicological mechanisms. However, a substantial translational gap remains between in vitro screening data and complex in vivo systemic effects. To address this limitation, researchers have integrated physiologically based toxicokinetic (PBTK) models, which mathematically incorporate in vitro-derived permeability coefficients into differential equations, enabling robust in vitro-to-in vivo extrapolation (IVIVE) for predicting systemic effects in humans [44,45]. For flame retardants such as decabromodiphenyl ethane, which exhibit extreme hydrophobicity and highly complex excretion kinetics, investigators have combined data mining, weight of evidence frameworks, and computational toxicology models (e.g., chemical activity analysis) to accurately quantify their bioaccumulation potential across aquatic and terrestrial food webs [46]. For more complex open-environment scenarios, such as reclaimed water irrigation, the strategic integration of riverine contaminant fate models with coupled soil–plant models enables the systematic prediction of the dynamic fate and health risks of ECs without requiring additional in vivo assays [47].

While the compilation of standardized, high-quality, and publicly accessible chemical toxicity databases is an essential prerequisite for developing robust predictive models [48], in silico approaches have become a foundational pillar for constructing proactive risk prediction frameworks when assessing the tens of thousands of industrial chemicals that lack empirical toxicological data [49]. This animal-free virtual screening approach excels at high-resolution toxicity prediction and offers high computational efficiency. A representative example is RespiraTox, a random forest model developed to predict human respiratory irritation, which successfully shifted the field from reliance on historical empirical observation to QSAR-based prediction [50]. Similarly, the CarcGC deep learning model uses graph convolutional networks to map molecular topological structures and can accurately identify potentially high-priority carcinogenic risks among hazardous air pollutants [51]. When assessing extreme chemical threats or emerging nanomaterials, in silico approaches deliver irreplaceable, game-changing disruptive value. Using phosgene oxime as a case study, which is a highly regulated, extremely toxic chemical warfare agent that lacks comprehensive empirical toxicity data, researchers utilized molecular structure fingerprints to conduct a holistic, quantitative evaluation of its acute toxicity and mutagenicity by integrating leading computational platforms, including STopTox, ADMETlab, and VEGA. This approach effectively overcomes the dual bottlenecks of ethical constraints and experimental safety hazards [52]. Similarly, by encoding intrinsic physicochemical properties of nanomaterials (e.g., core particle size and surface charge), machine learning algorithms, which are most notably support vector machines and artificial neural networks (ANN), can accurately predict the median lethal concentration (LC_50_) for aquatic invertebrates such as *Daphnia magna*, substantially reducing reliance on conventional aquatic bioassays [53]. Endorsed by the International Council of Chemical Associations and the U.S. EPA, read-across and in silico profiling are now recognized as core regulatory tools for filling toxicological data gaps [54]. This paradigm shift not only marks a profound advancement in toxicological research, transitioning from fragmented local observations to systematic integration, and from macroscopic biological endpoints to microscopic molecular mechanisms, but also establishes a scientifically rigorous, ethically sound, and highly efficient framework for the global prediction and regulatory governance of high-risk chemicals.

However, there are also certain limitations. For example, whilst some in vitro and cellular experiments are better suited to elucidating mechanisms of action, they tend to have relatively low throughput and are more costly; conversely, in silico methods (QSAR and machine learning) offer extremely high computational throughput and are best suited to large-scale virtual screening (such as for nanomaterials or regulated compounds where data are extremely scarce), but their predictive accuracy is highly dependent on the quality of the training data. When the training set lacks specific structural analogs, the predictive results of machine learning or read-across approaches often exhibit bias or even conflicting conclusions across the literature; it is therefore essential to utilize a weight of evidence (WoE) framework to mitigate the uncertainty arising from a single model or data limitations [53,55]. Future research should focus on bridging the gap in throughput and accuracy between in vitro experiments and computational predictions, establishing a closed-loop workflow of “computational prediction—in vitro validation—data feedback—model iteration”. This is expected to enable high-throughput, low-cost, and highly reliable alternative assessments whilst maintaining mechanistic insights.

### 4.3. Technological Evolution and Synergy in Human Health and Environmental Hazard Characterization

**Figure 9:**
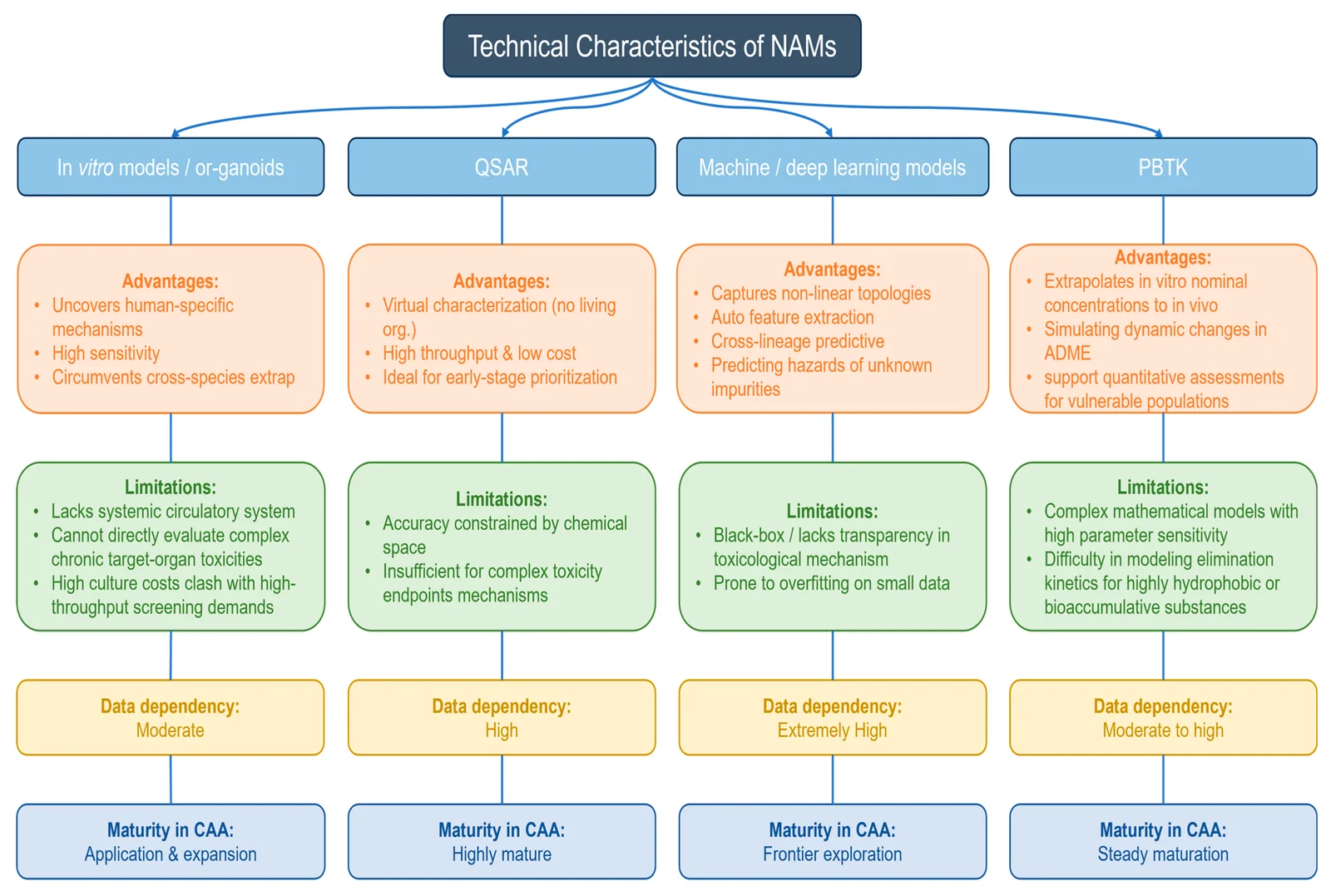
Horizontal comparison of technical characteristics of NAMs in chemical alternatives assessment.

In summary, hazard characterization is undergoing a profound paradigm shift, transitioning from conventional in vivo animal bioassays toward mechanism-driven and data-centric predictive modalities, collectively termed NAMs. To clearly delineate the operational scope of these emerging tools within CAA, this study systematically reviews core NAM modalities, including in vitro organoid models, QSAR modeling, machine/deep learning algorithms, and PBTK modeling. Furthermore, a lateral multi-criteria comparison is conducted across four fundamental metrics of core advantages, primary limitations, data dependency, and regulatory readiness within the current CAA framework, with the results summarized in Figure 9.

Converging demands from modern chemical safety governance and pharmaceutical research and development for high-throughput assessment, proactive hazard forecasting, and ethical compliance are accelerating the integration of human health and environmental hazard characterization into the NGRA framework. This evolution does not represent a simplistic abandonment of traditional experimental science, but rather a profound conceptual reframing: shifting inference from observational phenotype characterization toward mechanistic elucidation of hazard pathways. Systemic phenotypic validation, provided by conventional bioassays and high-throughput mechanistic prediction delivered by NAMs, converges organically and complements one another deeply, together forming the foundational cornerstone of the NGRA paradigm. This profound transition from an experiment-driven framework to a data-centric and mechanism-driven framework is both legally and ethically aligned with global regulatory trajectories prioritizing alternatives to animal testing. Of course, in the future, advanced NAM methods will also need to undergo rigorous standardization and validation by all international regulatory bodies; the shortcomings of high-throughput methods in predicting long-term, low-dose chronic synergistic toxic effects will also need to be addressed. Only in this way can a scientifically rigorous, efficient, comprehensive and ethically compliant global early-warning management system for the full life cycle of chemicals be established.

## 5. Multi-Scale Environmental and Human Exposure Assessment

**Figure 10:**
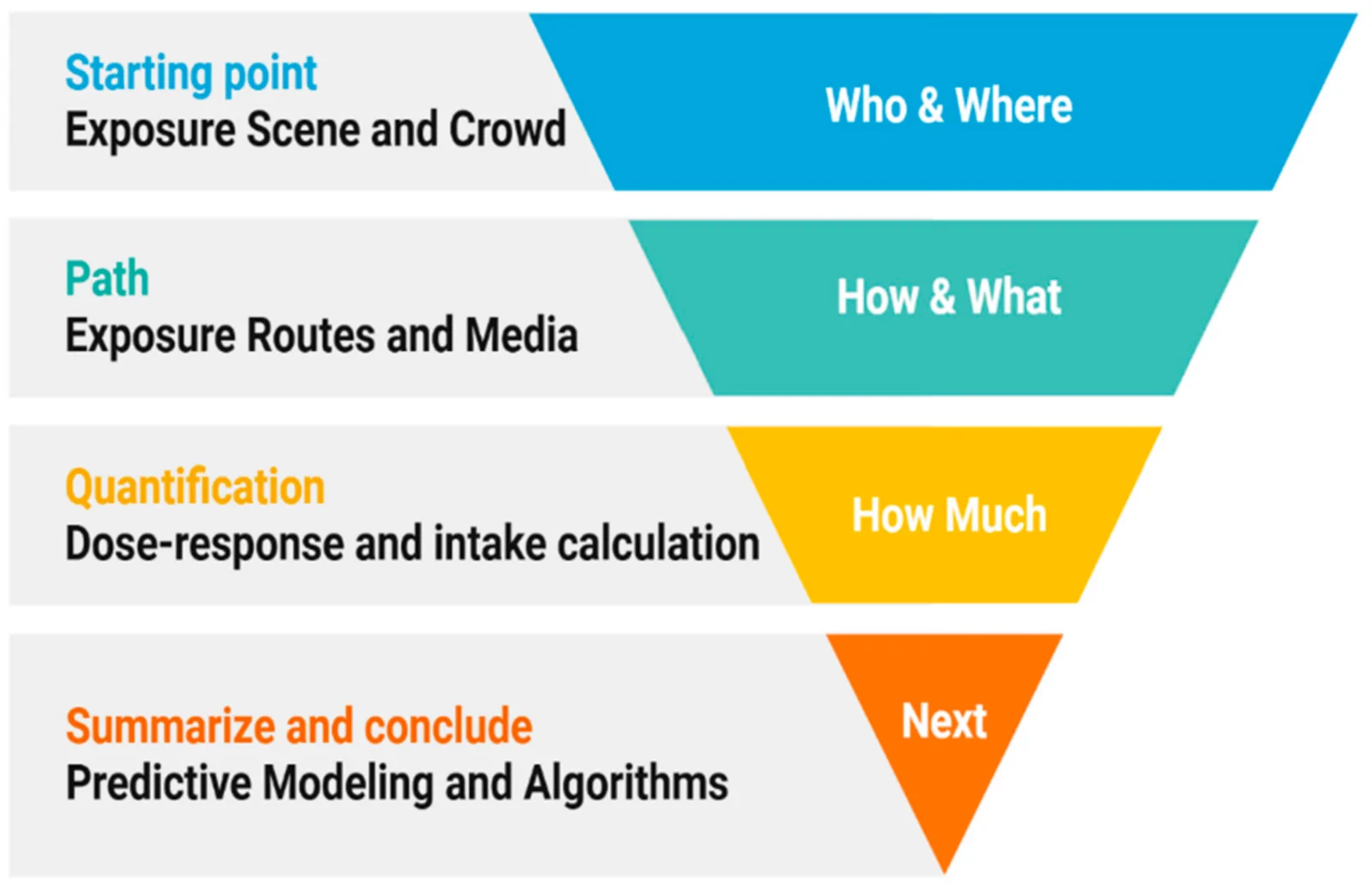
Workflow of exposure assessment.

Figure 10 systematically illustrates the step-by-step workflow of exposure assessment, covering the complete process from macro-level qualitative screening to micro-level quantitative measurement, and subsequently to active predictive modeling. At present, accurate characterization of the actual exposure levels of chemical substances in human populations and environmental compartments remains an essential prerequisite for conducting a rigorous environmental health risk assessment and implementing robust chemical safety governance.

As the foundation of exposure science, conventional exposure evaluation models have established the fundamental physical logic of “exposure pathway × contact rate”. These frameworks mostly rely on simple physicochemical properties, field monitoring data, and empirical models to estimate human exposure potential or conduct quantitative risk analyses. For instance, traditional environmental epidemiological models have been widely used to assess non-carcinogenic and carcinogenic risks linked to dietary arsenic intake in specific populations, where the standard hazard quotient and cancer risk metrics are applied to quantify corresponding toxicological potential [56]. Similarly, other researchers have adopted urinary biomarkers to monitor daily intakes of BPA and its alternatives, and compared measured values with established health benchmark values [57]. However, conventional models are generally constructed under hyper-conservative “worst-case scenario” assumptions. Although this one-size-fits-all, conservative, and static assessment approach has successfully protected the baseline of environmental safety, it inevitably leads to substantial overestimation of actual risks in the current context of rapidly expanding chemical inventories and highly dynamic exposure scenarios. As a result, this traditional paradigm often causes promising alternative chemicals to be disqualified prematurely during initial screening, revealing a fundamental structural contradiction defined by low throughput, high time costs, and a clear decoupling between analytical efficiency and assessment accuracy.

### 5.1. Refinement and Dynamism in Exposure Assessment

To correct for systemic biases inherent in conventional models, researchers have developed high-resolution individualized model refinements without modifying the underlying mechanistic physical framework by incorporating stochastic formulations, multi-media spatial coupling, and internal physiological compartmental mechanisms. As a result, rigid and worst-case upper-bound deterministic assumptions have been successfully replaced with dynamic probability distributions for key exposure parameters. Within this pragmatic program of refined optimization, multi-pathway algorithms and cumulative risk assessment frameworks lead the ongoing methodological paradigm shift. Multi-pathway algorithms overcome the fragmented, single-pathway calculations that constrained prior work by seamlessly integrating three non-dietary exposure pathways, including dust ingestion, dermal contact, and inhalation. They systematically quantify holistic human exposure risks from flame retardants and plasticizers in indoor environments [58]. Concurrently, cumulative risk assessment models are designed to address co-occurring pollutants that share common toxicological mechanisms, such as phthalates and their structurally diverse alternative plasticizers [59]. Using deterministic exposure/risk equations and multiple linear regression, and following a transparent process comprising ‘equation—parameterization—normalization—comparison with reference standards’, these models are capable of quantifying individual exposure levels to a variety of chemicals, identifying compounds to which individuals are simultaneously exposed, and providing baseline data for assessing the potential health risks of combined exposure.

Furthermore, researchers have progressively improved the precision of exposure science through rigorous confounding factor control, spatial scale optimization, and customized targeted parameterization. By leveraging large-sample cohorts and constructing multiple linear regression models, prior work has successfully adjusted for lifestyle confounders, including smoking status, age, and body mass index, enabling precise isolation of the “occupational contribution” of electronic waste processing to population exposure burdens of alternative plasticizers [60]. Concurrently, for assessments at hazardous waste sites, researchers have developed highly granular customized parameters by characterizing contamination enrichment across discrete particulate matter size fractions and accounting for physiological and behavioral differences across population subgroups. This customized parameterization substantially improves the analytical resolution and accuracy of vulnerability assessments for susceptible populations [61]. These refinements have also been extended through deeper integration of physiological processes and expansion to incorporate multi-media ecological coupling. Researchers have not only developed a core human physiologically based toxicokinetic (PBTK) model using clinical data from human volunteers that can simultaneously simulate absorption, distribution, metabolism, and excretion (ADME) profiles for four major classes of environmental phenols, enabling a critical advance from modeling external exposure loads to estimating internal target doses in specific organs [62], but also constructed a joint multi-media evaluation framework (water–sediment–soil–biota) specific to per- and polyfluoroalkyl substances (PFAS). By using mosses as natural bioamplifiers to characterize complex exposure pathways in enclosed wetland systems and grounding analyses in updated toxicokinetic dose calculations, this framework rigorously ensures that comprehensive assessments remain both temporally relevant and fit for regulatory decision-making [63]. However, many new alternative chemicals lack basic physicochemical parameters (such as the octanol–water partition coefficient and degradation half-life), which means that the application of high-resolution dynamic exposure models is often constrained by a lack of underlying experimental data.

Overall, while conventional exposure models remain valuable as cost-effective preliminary screening tools to rapidly rule out low-risk substances, the optimized and refined model configurations are specifically tailored for borderline-risk chemicals, structurally complex compounds, or substances with high economic stakes, enabling high-precision and quantitative risk evaluation. Although these refined iterations do not fundamentally overhaul the underlying mathematical paradigms of conventional models, they substantially unlock the latent potential of established methodologies. This strategic improvement therefore represents a key approach to support high-resolution risk assessment focusing on vulnerable populations and high-priority exposure pathways [64].

### 5.2. Novel Exposure Assessment and Multi-Media Predictive Modeling

Although minor modifications to traditional models have gradually improved analytical accuracy, within the CAA framework, these methods remain fundamentally reliant on existing observational datasets. Faced with tens of thousands of alternatives, traditional monitoring methods have proven inadequate due to a lack of experimental toxicity and exposure data. It is therefore necessary to employ novel computational models centered on QSAR, machine learning, and IVIVE for high-throughput screening. To fill critical data gaps through the integration of NAMs, a series of new alternative assessment strategies has been developed to address hundreds of unknown impurities and substances lacking toxicological reference values, particularly non-intentionally added substances in food-contact materials. These pioneering frameworks seamlessly integrate estimated daily intake models with the threshold of toxicological concern approach, computational modeling, and in vitro bioassays. By leveraging big data and advanced algorithmic processes to “predict the unknown”, this comprehensive paradigm effectively dismantles the traditional regulatory misconception of “no data, no risk” [65].

To circumvent the methodological limitation of overreliance on animal surrogates for establishing safety margins, IVIVE frameworks have been developed to integrate human in vitro assay data with multi-scale computational kinetic simulations. On one hand, this integrated paradigm successfully extrapolates nominal in vitro concentrations into human-relevant safety metrics, such as tolerable daily intakes and biomonitoring equivalents tailored for pregnant populations [66]. On the other hand, it enables reverse dosimetry via reverse toxicokinetic models, leveraging internal exposure profiles from neonatal serum to retrospectively estimate actual maternal and environmental intake rates [67]. PBTK/IVIVE-based reverse dose estimation models are best suited to scenarios where internal biomonitoring data are available but external environmental exposure concentrations are lacking, as they enable the accurate extrapolation of internal exposure doses.

When dealing with the complex challenge of multi-component chemical mixtures in real-world human exposure scenarios, these new computational models also demonstrate remarkable capabilities, enabling the integration of multi-media environmental aggregation with high-throughput LCA. By incorporating weighted quantile sum regression and Bayesian kernel machine regression, these advanced statistical frameworks can not only quantify the combined cumulative effect of complex multi-component mixtures on the incidence risk of congenital hypothyroidism, but also accurately estimate the relative contribution of each individual chemical component [68]. Moreover, by leveraging coupled multi-media environmental aggregation and exposure characterization models (e.g., USEtox) embedded in the hierarchical assessment framework, researchers can transition seamlessly from qualitative scanning to higher-order quantitative analysis, thereby providing critical forward-looking guidance for the sustainable life cycle design of next-generation perovskite-based devices [69].

To catalyze the paradigm shift in consumer product chemical management toward “eco-effectiveness”, the institutionalization of high-throughput exposure assessment frameworks rooted in an absolute sustainability perspective is urgently needed. This computational architecture, which integrates coupled near-field and far-field exposure models with mass-balance-based matrix methods, enables researchers to holistically evaluate life cycle exposures across diverse population cohorts. In turn, this approach facilitates high-throughput quantitative prioritization of thousands of chemical alternatives in cosmetics and personal care products [69]. Notably, analogous coupled frameworks using the product intake fraction metric have been successfully implemented to screen chemicals of concern in plastic toys [70].

At the operational level, this proactive approach is applied across screening-level hazard characterization scoring, product-specific exposure simulation, and semantic web ontology modeling. Researchers not only use QSAR tools, such as EPI Suite and the OECD QSAR toolbox, to conduct multi-media simulations for emerging chemicals, leveraging the Ruler system for lateral benchmarking against reference datasets to achieve high-throughput prioritization [71], but also develop mathematical algorithms within the ConsExpo web platform to simulate multi-cohort pediatric exposure dynamics, effectively addressing long-standing data gaps related to powder inhalation risks across different childhood age groups [72]. Furthermore, to overcome the labor-intensive bottleneck of life cycle data collection, researchers have developed a semantic web ontology framework to link LCA with human exposure modeling. By engineering the “ExoMap” bridging ontology to enable automated machine querying (via SPARQL queries) and rapid parameterization, this paradigm enables instantaneous exposure modeling while eliminating costly manual literature curation [73].

The core value of computational prediction models lies in their ability to advance chemical evaluation to the molecular design stage and to address data-sparse domains. QSAR and machine learning models are best suited to early-stage virtual screening, where data are extremely scarce and compound libraries are vast. Although some advanced models still require final calibration against empirical datasets to meet requirements for absolute accuracy and biological mechanistic validation, their unparalleled high-throughput capability and macro life cycle perspective nonetheless impart bulk CAA with a proactive vision that is inherently unattainable via conventional methodologies.

Although methods such as the automated extraction of data using the Semantic Web (ExoMap) and complex deep-learning-based multi-media dynamic simulations offer extremely high throughput, their algorithmic generalization capabilities and the extent of end-to-end validation remain in the exploratory stage within regulatory science and have not yet been fully adopted as statutory standards by all international regulatory bodies. Future research should focus on enhancing the cross-scenario generalization capabilities of these models, establishing standardized end-to-end validation frameworks, and promoting collaboration between regulatory bodies, academia, and industry to develop universally accepted statutory standards, thereby accelerating their transition from research tools to regulatory applications.

### 5.3. Synergistic and Complementary Relationships Among Three Generations of Models

**Figure 11:**
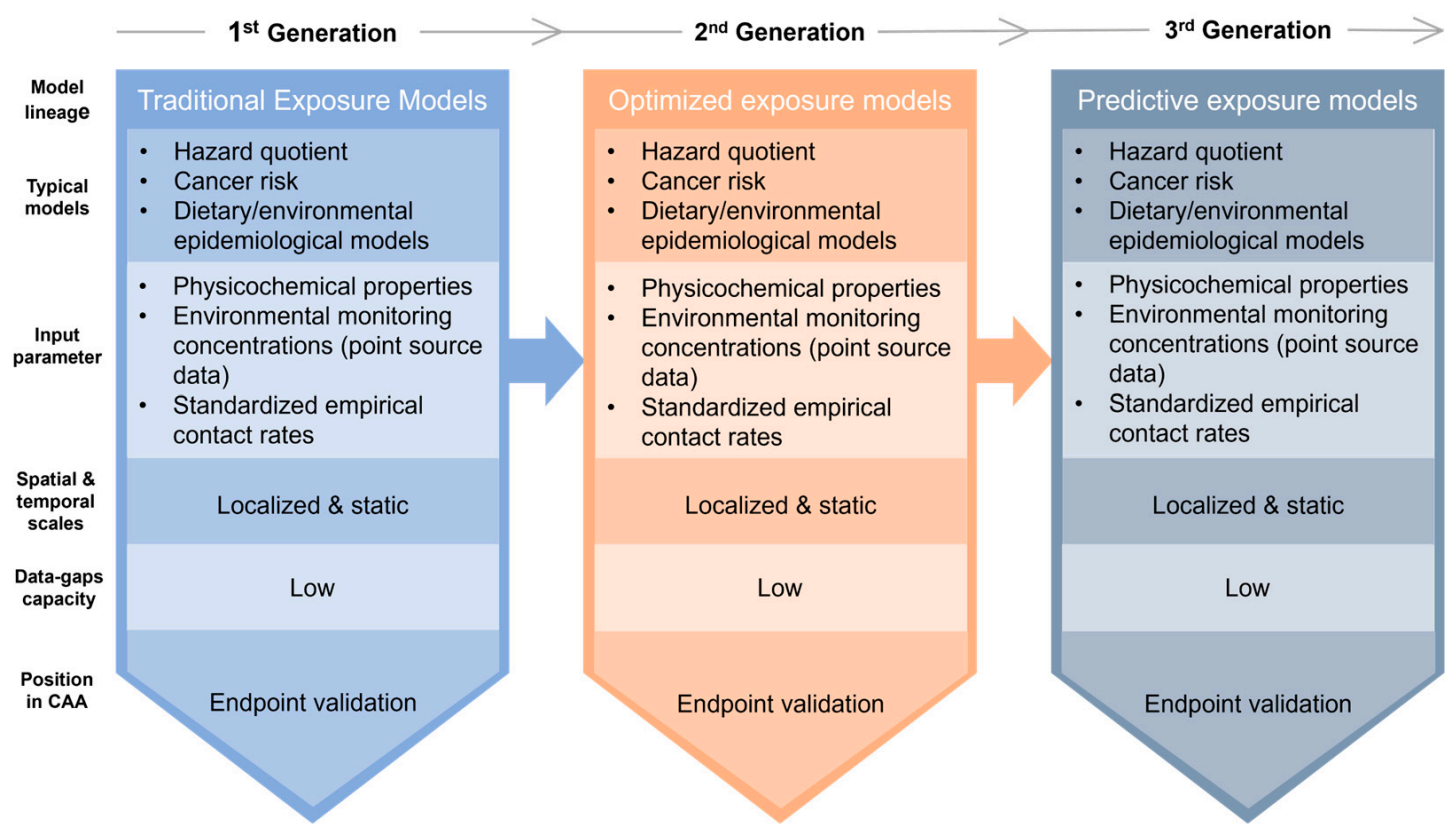
Comparison of technical parameters and gaps-filling capacities among three generations of environmental and population exposure models.

In summary, these three generations of exposure assessment models, specifically conventional, refined, and novel frameworks, are by no means mutually exclusive competing approaches or absolute replacements in modern environmental governance systems. Instead, they constitute a progressive developmental continuum, characterized by mutually supportive underlying data foundations, hierarchically structured assessment scales, and deeply complementary application scenarios. To synthesize their synergistic and complementary mechanisms more intuitively in chemical life cycle substitution processes, this section systematically reviews the differences across the three generations in terms of input parameter requirements, spatiotemporal resolution, and the ability to address gaps in empirical data, which allows us to explicitly define their specific functional roles within the overall CAA workflow (Figure 11).

Accordingly, future research and regulatory applications must move beyond the narrow focus on individual technological iterations and instead prioritize the seamless integration of these three modeling paradigms. In this proposed integrated workflow, novel simulation frameworks are first applied to conduct high-throughput reverse dose–response analysis and early-stage risk prediction, enabling rapid screening and prioritization of large numbers of candidate chemicals. Next, refined conventional methods, which incorporate more detailed parameters and explicit internal physiological mechanisms, are used to identify key exposure pathways and characteristics of susceptible populations. Finally, established biomonitoring and environmental monitoring approaches are utilized to provide definitive endpoint validation. This progressive, multi-source data fusion framework will substantially improve the scientific rigor, timeliness of response, and proactive judgment of modern CAA and environmental risk management.

However, when discussing the synergy and complementarity of the three-generation models, we must also acknowledge an objective reality: in the existing literature, safety thresholds derived from different models for the same emerging alternative often contradict or diverge. This conflict in scientific understanding highlights the need to adopt a ‘Weight of Evidence’ (WoE) integrative framework, which is a key consideration for future research.

## 6. LCA Extending from Source to Sink

**Figure 12:**
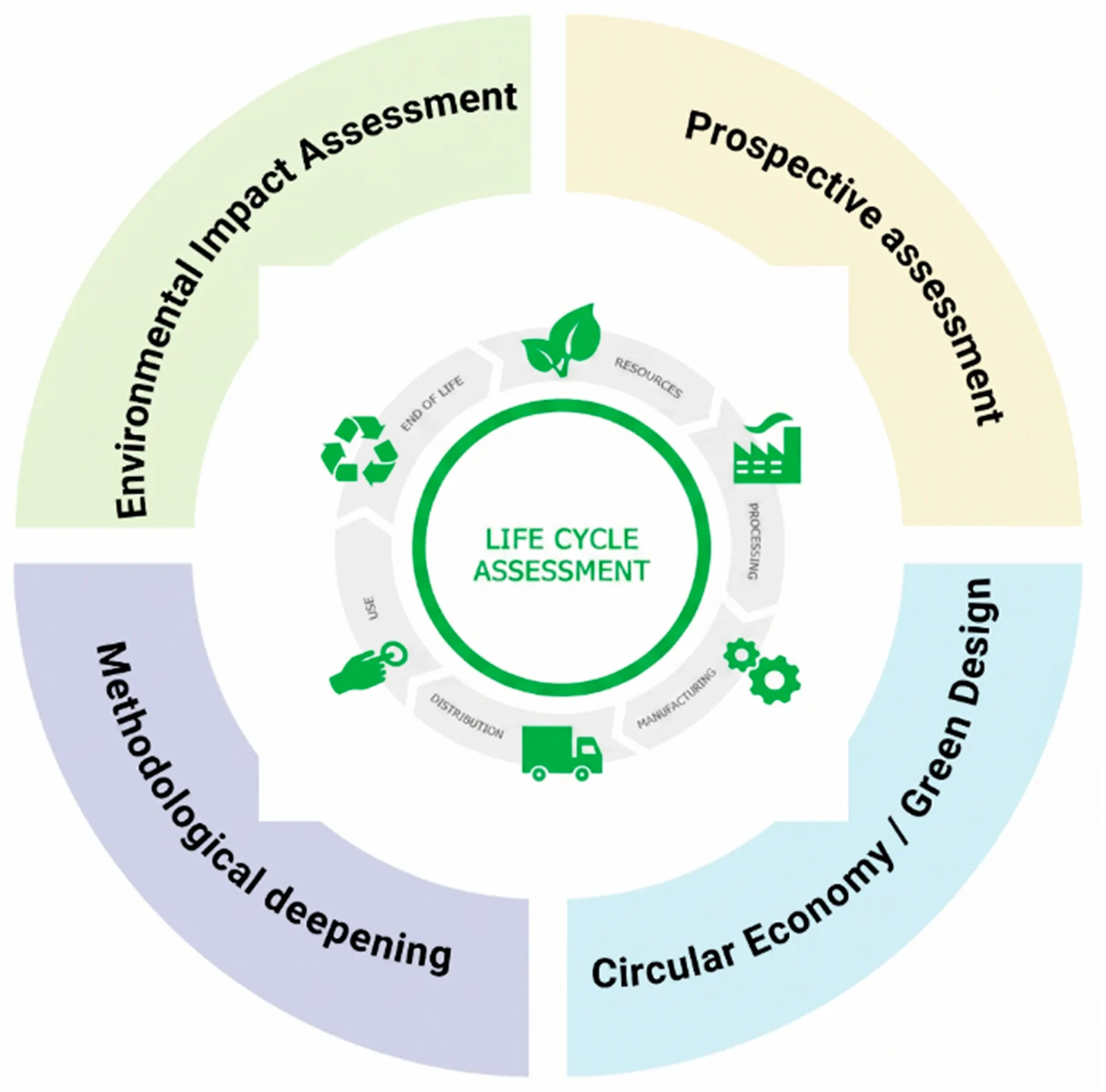
Life cycle assessment process and evolutionary concepts.

LCA, a systemic and full-supply chain evaluation tool that accounts for multiple impact indicators, has progressively emerged as the cornerstone of sustainable CAA. As illustrated in Figure 12, the centralized life cycle process forms the core data substrate for quantifying environmental impacts, while the four peripheral research dimensions further expand the analytical boundaries and application scenarios of conventional LCA frameworks. Although classical life cycle inventory (LCI) data remain the foundation of the entire evaluation framework, LCA applications in the chemical sector have extended far beyond routine environmental impact reporting. Instead, the field has advanced inward to catalyze circular economy transition and green product/process design, expanded outward to develop prospective assessments tailored for emerging future technologies, and continuously driven methodological advancement to address empirical data gaps and resolve model coupling constraints.

### 6.1. Environmental Impact Assessment

As a classical cornerstone of industrial ecology, life cycle impact assessment (LCIA) plays an indispensable role in CAA, expanding analytical boundaries to encompass the full supply chain sequence from raw material extraction to final waste disposal. Currently, conventional LCA predominantly focuses on process-level inventory auditing and environmental performance quantification of mature industrial pathways. On one hand, existing investigations of critical green chemical platform chemicals, such as bio-based succinic acid derived from non-food crops, demonstrate exemplary low-carbon and energy-saving performance, with substantially reduced global warming potential (GWP) (the LCI databases are Ecoinvent and US LCI; the software is SimaPro; the allocation principle is “System expansion”). These findings provide pre-construction quantitative emission-reduction benchmarks for the chemical industry [74]. On the other hand, rigorous systems auditing of conventional agriculture and biomass supply chains reveals hidden environmental deficits. For example, the systemic environmental burden associated with wheat straw pulp production in China is significantly higher than that of conventional wood pulp, primarily driven by extensive coal combustion and the use of bleaching chemicals (the LCI database is Ecoinvent; the software is GaBi; the allocation principle is “ Cut-off”) [75]. Similarly, for sisal yarn production in Tanzania, pesticide and chemical fertilizer application during the agricultural stage accounts for the vast majority of the product’s total ecotoxicological impacts (the LCI database is Ecoinvent 3; the software is SimaPro 9.6.0.1; the allocation principle is “Cut-off, Unit approach”) [76].

Overall, current LCA-based environmental impact evaluations are fully capable of providing relatively objective, multi-dimensional quantitative datasets to support chemical substitution decisions. Although these traditional “retrospective” and “static” assessment approaches precisely quantify environmental hotspots for existing technologies, their heavy reliance on inventories from commercialized, mature industrial systems inevitably introduces temporal mismatch and data lag when evaluating novel, non-commercialized green alternatives, which ultimately reduces the accuracy of evaluation results. This systemic limitation highlights the critical need for a paradigm shift in the field toward the development of more predictive, prospective technology assessment frameworks.

### 6.2. Prospective Evaluation

With the explosive advancement of green chemistry and synthetic biology, many novel chemical alternatives in early-stage research, which are characterized by low Technology Readiness Levels (TRLs), have demonstrated substantial ecological potential at the laboratory scale. A core function of Prospective LCA is to conduct predictive modeling for future scenarios before technologies are scaled to industrial production, thereby resolving the well-documented “Collingridge dilemma” inherent in the environmental assessment of emerging technologies [77]. Current research trajectories in this field are highly diverse. Most commonly, researchers use process engineering calculations and scaling models to extrapolate bench-scale laboratory data (TRL 3–5) to future full industrial scales (TRL 9). For instance, integrating future electricity grid projections from Shared Socioeconomic Pathways (SSPs) into production forecasts for cellulose nanocrystals (CNCs) and bio-based adipic acid allows researchers to proactively identify hidden energy-intensive sinks and material conversion bottlenecks prior to commercial-scale manufacturing [78]. This methodology is particularly effective for anticipating industrialization barriers for emerging materials. For instance, it has been used to pre-calculate the severe energy penalties associated with scaling graphitic carbon nitride (g-C_3_N_4_) photocatalysts from gram-scale to ton-scale production [79], and to conduct comprehensive auditing of greenhouse gas emissions linked to palm oil sourcing and transoceanic transport for poly(3-hydroxybutyrate-co-3-hydroxyhexanoate) (PHBH) during its upscaling phase [80]. Critically, this approach can even function as an early preemptive screening tool. Research has demonstrated that the “biogenic green synthesis” of silver nanoparticles, despite strong performance at the laboratory scale, would paradoxically result in a “regrettable substitution” upon industrial scaling due to low separation efficiency [81].

To standardize these predictive tools and address the historical under-evaluation of novel leather alternatives for the footwear industry, hybrid evaluation frameworks that integrate qualitative life cycle screening (LiSET) with simplified quantitative LCA have been developed. These frameworks enable early-stage fine-tuning of technological development pathways by predicting industrial performance during pilot-scale testing phases [82]. Prospective LCA effectively embeds environmental considerations at the earliest stage of chemical research and development, providing valuable predictive insight to mitigate early-stage investment risks and avoid suboptimal technological development. Because Prospective LCA relies heavily on scenario-based assumptions, its assessment outcomes inherently exhibit wide confidence intervals and lack comprehensive validation against actual industrial operating data. To improve the utility of these future-oriented projections, contemporary research is increasingly shifting toward aligning assessment focal points with concrete design parameters and closed-loop production systems, facilitating the deeper integration of LCA with circular economy and green design principles.

### 6.3. Circular Economy/Green Design

Unlike traditional environmental impact assessments that primarily focus on “problem diagnosis”, the deep integration of LCA into circular economy and green design practices facilitates a strategic paradigm shift toward “upstream intervention”. Within the framework of chemical alternative evaluation, true sustainability requires alternatives to exhibit low toxicity and integrate seamlessly into closed-loop industrial ecosystems. First, as a leading example in green design, hemp-based insulation materials demonstrate significant negative carbon benefits when assessed via bio-carbon accounting, a result of their strong carbon sequestration capacity during the cultivation phase [83]. Similarly, for heavy-duty transportation clean fuel alternatives, oxygenated methyl ethers (OMEs) use syngas produced via gasification of agricultural and forestry residues as their sole carbon source. By inhibiting the formation of soot precursors at the molecular level, OMEs establish a closed, well-functioning bio-carbon cycle [84]. Second, in the high-value reuse of agricultural and industrial waste, cross-sector applications of alternative materials deliver substantial ecological benefits. In the agricultural sector, the biodegradable polymer poly(butylene succinate-co-adipate) (PBAT) is synthesized from non-food biomass feedstocks such as corn stover (the LCI database is the Ecoinvent database, Version 3.8; the software is SimaPro 9.4; the allocation principle is “mass allocation”) [85]. Similarly, composite microbial fertilizers (CMF) processed from urban organic waste replace highly polluting conventional fertilizers (the LCI database is the GaBi database; the software is GaBi 10.0/GaBi 10.0.0.71; the allocation principle is “Cut-off”) [86]. Both examples effectively reduce GWP value and convert waste streams into high-value resources. In parallel, within the industrial sector, urban biowaste is converted to clean energy (methane or syngas) [87]; sulfur recovered from petroleum processing is converted into polymeric sulfur via reverse vulcanization, a product that exhibits excellent thermal tunability and eliminates the need for landfill disposal of excess sulfur [88]; and glycerol, a major byproduct of biodiesel manufacturing, is repurposed as a plasticizer for microalgae-based bioplastics [89]. All these cases achieve cross-sector collaborative valorization of waste streams.

Nevertheless, in the existing literature, there are often conflicting conclusions as to whether the high-value utilization or recycling of waste always yields a net environmental benefit. Systematic LCA analysis focused on the end-of-life (EOL) stage of alternative materials reveals the inherent complexities in their sustainability performance. Cross-linked polyethylene (PEX) pipes achieve up to a 63% cost reduction over their full life cycle compared to conventional copper pipes, alongside substantial improvements in GWP and carcinogenic risk [90]. However, another widely promoted alternative material, e.g., fiber-reinforced polymer (FRP) composites, presents a distinct sustainability paradox. While FRP delivers clear green design benefits via light weighting for automotive and pipeline applications, its upstream raw material production remains highly energy-intensive, and the material faces systemic barriers due to EOL recycling difficulties [91].

LCA has evolved into an indispensable tool for quantifying the economic performance of circular systems and evaluating the effectiveness of green design innovations. It effectively dispels the pervasive misconception that “recycling equals green” and provides robust data-driven evidence. Poorly designed recycling pathways can generate higher net environmental burdens than conventional linear production systems. However, for both mapping complex circular value chains and simulating prospective sustainability scenarios, current LCA methodologies remain significantly limited in their ability to characterize toxicological mechanisms and complex environmental behaviors specific to individual chemical substances. This methodological gap highlights the critical need for transformative, fundamental innovation in LCA’s core analytical framework.

### 6.4. Methodological Deepening

When confronting the vast universe of chemical entities and highly convoluted environmental exposure mechanisms, conventional LCA frameworks face fundamental challenges of acute empirical data gaps and a substantial misalignment between characterization models and real-world toxicological mechanisms. This bottleneck has catalyzed interdisciplinary efforts to advance the methodology toward refined, mechanistically driven modeling. On one hand, the integration of machine learning and big data has effectively addressed blind spots in historical data. Emerging workflows such as LCA implementations built on predictive tools like the CLICC framework use molecular structure-based ANNs to estimate supply chain carbon dioxide emissions and cumulative energy demand from only chemical molecular formulas, while introducing “functional equivalence factors” to quantify the actual mass of chemical required for a given functional unit [92]. Furthermore, by developing an intelligent agent system that integrates chemical engineering thermodynamic models with LCA inventories, researchers have successfully optimized the integrated production process for high-value-added bio-lubricants. This approach overcomes the inherent latency of traditional LCA and eliminates the limitation of being unable to support real-time engineering design iterations [93]. On the other hand, the cross-disciplinary integration of environmental toxicology and precision exposure science has elevated the scientific rigor of toxicity quantification in LCA. Amid the ongoing phase-out of hazardous PFAS within the outdoor apparel industry, a novel LCA framework has leveraged toxicokinetic datasets to extrapolate epidemiological human exposure models for short-chain fluorinated PFAS alternatives. This development successfully resolves long-standing calculation blind spots associated with characterization factors for persistent, mobile, and toxic substances [94].

Ultimately, this methodological deepening is driving a paradigm shift in LCA from macroscopic and descriptive statistics toward high-fidelity and mechanistic modeling. When combined with advanced data imputation, robust toxicity characterization, and structured uncertainty management, this framework substantially improves data quality and model reliability. In turn, it strengthens the empirical foundation required for the environmental impact assessments, prospective forecasting, and circular economy design activities.

### 6.5. Toward Life Cycle Systems Anchored in Planetary Boundaries

**Figure 13:**
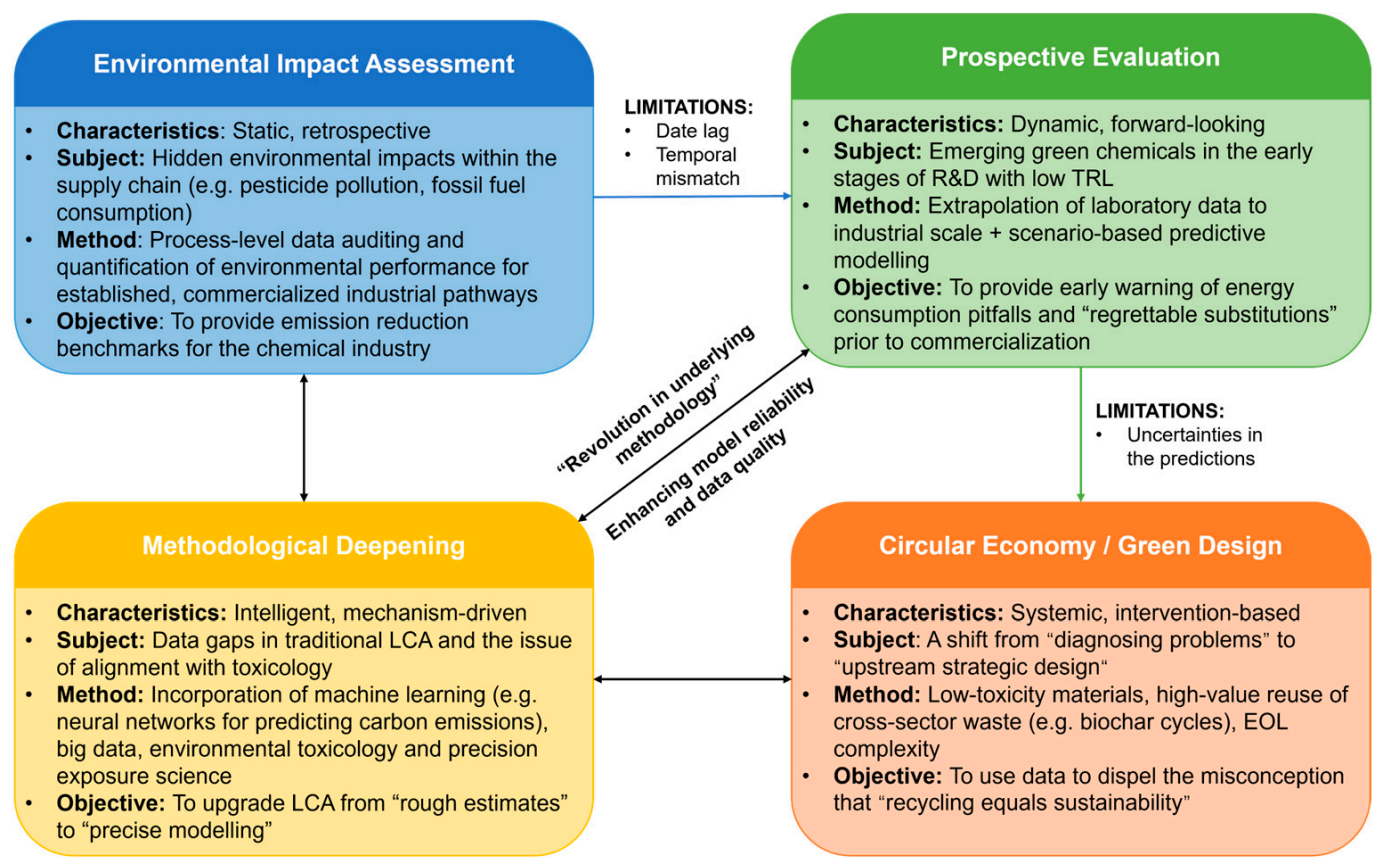
Four-dimensional extension of LCA and mechanisms of interaction.

From a global perspective, the unregulated production and consumption of chemicals classified as “novel entities” have already exceeded the safe operating space of the Earth’s system [95]. The integration of LCA into chemical alternative evaluation represents a significant paradigm shift from passive end-of-pipe toxicity control toward a systematic early warning framework. To bridge the gap between the microscopic properties of chemical alternatives and macroscale ecological carrying capacities, integrating LCA within an absolute environmental sustainability assessment framework is an imperative step [96]. By synthesizing the four distinct research frontiers discussed above, including LCIA, prospective evaluation, circular economy/green design synergy, and methodological deepening (Figure 13), the integrated LCA framework effectively identifies and mitigates risks associated with “regrettable substitution”, while successfully shifting the assessment window to the earliest stage of molecular design and early-phase research and development. Through continued methodological refinement and cross-disciplinary collaboration, the life cycle paradigm will ultimately complete its transformation from a retrospective auditing tool to an upstream design roadmap, thereby establishing a robust empirical foundation for a safe, green, and truly sustainable chemical transition. This represents a functional extension of traditional chemical risk assessment methods, taken from a macro and systematic perspective, within the framework of CAA.

## 7. Conclusions

Traditional models, conventional exposure assessments. and classical LCA constitute a standard ‘toolkit’ for regulatory risk assessment; their core function is to answer the question: ‘Is a particular chemical substance safe?’ The integration of new multi-media computational models (such as USETox and the coupling of PBPK with exposure models) with NAMs (such as in vitro models, QSAR, and machine learning), however, marks a shift from “single-substance threshold determination” to a systematic assessment involving “multi-media, multi-route, full life-cycle and multi-alternative comparisons”. This represents a concrete application demonstrated and operationalized within the CAA framework. CAA does not seek to answer the question “Is a particular substance safe?”, but rather “Among several functionally equivalent alternatives, which one is relatively safer and more sustainable for humans and the environment across the entire life cycle and under different exposure pathways?”.

CAA, as the core enabling framework for avoiding “regrettable substitution” and advancing green chemistry, has undergone a fundamental shift from the conventional “one-to-one” drop-in substitution paradigm centered on single toxicity benchmarks to a multi-dimensional and system-wide integrated assessment. Current evaluative frameworks increasingly converge human and environmental hazard characterization, multi-scale exposure modeling, and LCA to break down historical methodological silos. This integration establishes a comprehensive and closed-loop assessment paradigm that spans from microscopic toxicological mechanisms to macroscopic ecological outcomes. The primary finding of this evolution is that cross-scale integration—linking microscopic toxicological mechanisms, mesoscale environmental transport, and macroscopic ecological outcomes—enables a more comprehensive evaluation of chemical substitution pathways. However, this integration is still at an exploratory stage and requires further methodological validation. Looking forward, future CAA must evolve from rigid evaluation into a dynamic, predictive “GPS” powered by big data and multi-scale mechanisms. This proactive approach will steer chemical development away from high-risk structural alerts, ultimately guiding academia and industry to implement SSbD frameworks that balance industrial performance, ecological safety, and low-carbon sustainability.

## Figures and Tables

**Table 1:** Guidelines for chemical alternatives assessment frameworks.

Organizations	Name	Reference
Toxics Use Reduction Institute	Alternatives Assessment Process Guidance	[10]
Lowell Center for Sustainable Production	Alternatives Assessment Framework from the Lowell Center for Sustainable Production	[11]
United Nations Environment Programme	General Guidance on Considerations Related to Alternatives and Substitutes for Listed Persistent Organic Pollutants and Candidate Chemicals	[12]
Umweltbundesamt	Guide on Sustainable Chemicals	[13]
United States Environmental Protection Agency	Design for the Environment Program Alternatives Assessment Criteria for Hazard Evaluation	[14]
National Research Council	A Framework to Guide Selection of Chemical Alternatives	[15]
European Chemicals Agency	Guidance on the Preparation of an Application for Authorization	[16]
Organization for Economic Cooperation and Development	Guidance on Key Considerations for the Identification and Selection of Safer Chemical Alternatives	[17]
Chinese Society for Environmental Sciences	Technical guidance for the assessment of green alternatives	[18]
Interstate Chemicals Clearinghouse, IC2	Interstate Chemicals Clearinghouse Alternatives Assessment Guide Version 1.2	[19]

**Table 2:** Main assessment perspectives within chemical alternatives assessment frameworks.

Frameworks	Health Hazards to the Human Body	Environmental Hazards	Physicochemical Properties	Full Life Cycle Assessment	Exposure Assessment
TURI: Alternatives Assessment Process Guidance	Necessary	Necessary	Necessary	Not necessary	Necessary
Alternatives Assessment Framework from the Lowell Center for Sustainable Production Version 1.0	Necessary	Necessary	Not mentioned	Not necessary	Not mentioned
UNEP: General Guidance on Considerations Related to Alternatives and Substitutes for Listed Persistent Organic Pollutants and Candidate Chemicals	Necessary	Necessary	Not mentioned	Not necessary	Necessary
EPA: Design for the Environment Program Alternatives Assessment Criteria for Hazard Evaluation	Necessary	Necessary	Necessary	Not mentioned	Necessary
UBA: Guide on Sustainable Chemicals	Necessary	Necessary	Necessary	Necessary	Necessary
OECD: Guidance on Key Considerations for the Identification and Selection of Safer Chemical Alternatives	Necessary	Necessary	Not mentioned	Not Necessary	Necessary
ECHA: Guidance on the Preparation of an Application for Authorization	Necessary	Necessary	Necessary	Necessary	Necessary
CSES: Technical guidance for the assessment of green alternatives	Necessary	Necessary	Not mentioned	Not mentioned	Not mentioned
IC2: Interstate Chemicals Clearinghouse Alternatives Assessment Guide Version 1.2	Necessary	Necessary	Necessary	Necessary	Necessary

## Data Availability

No new data were created or analyzed in this study. Data sharing is not applicable to this article.
